# The Results of Unstable Intertrochanteric Femur Fracture Treated with Proximal Femoral Nail Antirotation-2 with respect to Different Greater Trochanteric Entry Points

**DOI:** 10.1155/2020/2834816

**Published:** 2020-03-28

**Authors:** Sharan Mallya, Surendra U. Kamath, Rajendra Annappa, Nithin Elliot Nazareth, Krithika Kamath, Pragya Tyagi

**Affiliations:** ^1^Department of Orthopaedics, Kasturba Medical College, Mangalore, Manipal Academy of Higher Education, Manipal, India; ^2^Department of Orthopaedics, Father Muller Medical College Hospital, Mangalore, India; ^3^Kasturba Medical College, Mangalore, Manipal Academy of Higher Education, Manipal, India

## Abstract

**Background:**

Proximal femoral nail antirotation-2 (PFNA-2) has been widely used to treat intertrochanteric fractures with varied outcomes in the previous studies. The entry point of the nail plays an important role in achieving acceptable reduction, stable fixation, and avoiding implant related complications. This study was proposed to determine the optimal greater trochanteric entry point for PFNA-2 in unstable intertrochanteric femur fractures.

**Methods:**

We conducted an observational study on 40 patients with unstable intertrochanteric fracture treated with PFNA-2 implant in a tertiary care hospital. The patients were grouped into two based on the entry point: group L for lateral and group M for medial entry. Randomization was carried out by assigning the patients to the group by alternate allocation. The quality of reduction, tip apex distance, Cleveland index, and all the complications were noted. The final follow-up was conducted at six months. The functional outcome was evaluated using modified Harris hip score. The data analysis was performed using Student's *t*-test, chi square test, and Mann–Whitney test. A *P* value below 0.05 was considered significant.

**Results:**

Forty patients with 20 patients treated with medial entry point were included in group M and 20 patients in group L with lateral entry point. The group L had an average tip apex distance of 20.53 and group M had 20.02 (*P*=0.8). The complication of screw back out was seen in 3 out of 4 patients with poor reduction in group L. As per the Cleveland index, 6 patients in each group had suboptimal position and 4 out of 6 patients in group L with suboptimal position had screw back out. The lateral cortex impingement was seen in 14 patients of group L and 6 patients in group M with significant comparison (*P*=0.01). Three patients in group L had varus collapse with screw back out. Also, none in group M (0.05). The average modified Harris hip score in group L at six months follow-up was 71.94 and 76.8 in group M (*P*=0.84).

**Conclusion:**

Overall, to achieve good quality of fixation and reducing damage to gluteus medius entry point for PFNA-2 should be 5 mm medial to the greater trochanter tip.

## 1. Introduction

Intertrochanteric femur fracture is more common among elderly patients with osteoporosis and surgically fixing the fracture has been the accepted method to gain reduction and early mobilization [1]. Literature suggests that intramedullary nailing is one of the best choices for surgical fixation and has better clinical outcomes when compared to arthroplasty [2–4]. Proximal femoral nail antirotation-2 (PFNA-2) is the newer design and has been widely used to treat this fracture [5]. The results obtained with this implant in previous studies had varied outcome [6, 7]. This may be attributed to many factors like old age, fracture type, implant design, quality of reduction, and fixation. The entry point plays an important role in acceptable reduction, stable fixation, and avoiding implant-related complications [8, 9]. It has been suggested in a study that lateral entry point causes damage to the gluteus muscle tendon while reaming of intramedullary nail insertion. The study on anatomy of greater trochanter has concluded that entry point should be at the rear tip to accommodate the implant in proximal femoral medullary canal curvature [10]. This study was proposed to determine the optimal greater trochanteric entry point for PFNA-2 in unstable intertrochanteric femur fractures. The study was accepted by Institutional Ethics Committee.

## 2. Materials and Methods

**Figure 1:**
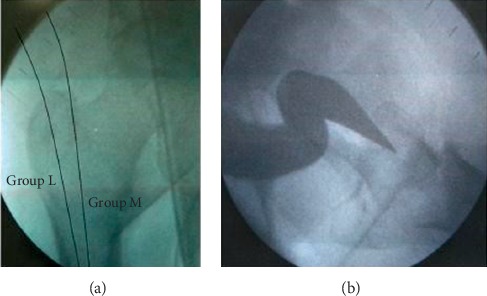
Entry points over the greater trochanter.

An observational study was conducted on 40 patients with intertrochanteric femur fracture operated with PFNA-2 implant in the Department of Orthopaedics, Government Wenlock Hospital, Kasturba Medical College, and its allied hospitals in Mangalore between January 2017 and July 2018. Patients with Type A31A2 and A31A3 (unstable intertrochanteric femur fracture) as per AO classification [11] and age more than 50 years were included. Patients with a stable type of fracture, expired before final follow-up, less than 50 years, and previous implant in the injured hip were excluded. The patients were divided into two groups: group M for medial entry (5 mm medial to the greater trochanter tip) and group L for lateral entry point over greater trochanter based on anteroposterior view of the X-ray. On lateral view of the X-ray, the entry point was in the centre (Figure 1). Randomization was carried out by assigning the patients to the group by alternate allocation.

The quality of reduction was noted by taking the difference of neck shaft angle between operated and normal hip. The difference of less than 5° was graded as excellent, between 5 and 10° as good, and >10° as poor [12]. The quality of fixation was evaluated using tip apex distance (T-A distance) [13]. The position of the compression screw was noted using the Cleveland index [14]. To avoid bias, the values were measured using MB ruler in the hospital's computed radiographic system by two trained orthopaedic surgeons.

All the complications were noted. Final follow-up was conducted at 6 months with modified Harris hip score (HHS) [15]. The study was permitted by the Institutional ethics Committee.

### 2.1. Data Analysis

The results were assessed using Student's *t*-test, chi square test, and Mann–Whitney test. A *P* value less than 0.05 was considered significant. The results were entered in MS Excel spreadsheet, and statistical analysis was performed using Statistical Package for Social Sciences (SPSS) version 16.0.

## 3. Results

**Figure 2:**
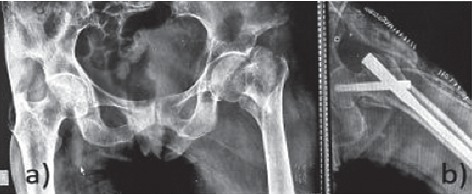
PFNA-2 with medial entry point.

**Figure 3:**
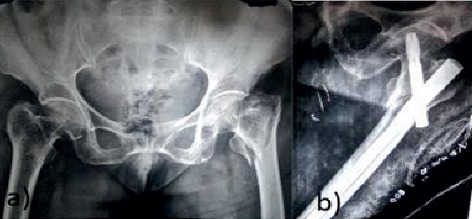
PFNA-2 with lateral entry point.

Forty patients with unstable intertrochanteric femur fracture, operated with PFNA-2, were enrolled in the study. Twenty patients treated with lateral entry point were included in group L (Figure 2), and group M included 20 patients with medial entry point (Figure 3). The average age of the patients in group L was 69.6 and group M was 69.85 (Table 1). The difference in age distribution was not significant between two groups (*P*=0.23). The group L had 12 female and 8 male patients, whereas group M had 8 female and 12 male patients (*P*=0.1). As per AO classification, group L had 13 patients with A2 type and 7 patients with A3 type. Group M had 11 patients with A2 type and 9 patients with A3 type of fracture (*P*=0.37) (Table 1).

### 3.1. Radiographic Parameters (Table 2)

The group L had average tip apex distance of 20.53 and group M had 20.02 (*P*=0.8). 4 in group L and 5 in group M had tip apex distance >25 mm (*P*=0.5) (Table 2). 3 out of 4 patients in group L with TAD more than 25 mm had screw back out. 3 patients of group L and 6 patients of group M had good reduction (5–10°) as per the neck shaft angle difference. 4 patients of group L had poor reduction (>10°). There were no patients with poor reduction in group M (*P*=0.08) (Table 2). The complication of screw back out was seen in 3 out of 4 patients with poor reduction in group L. As per the Cleveland index, 6 patients each in both groups had suboptimal position (*P*=0.63) (Table 2). Four out of 6 patients in group L with suboptimal position had screw back out.

### 3.2. Complications (Table 3)

**Figure 4:**
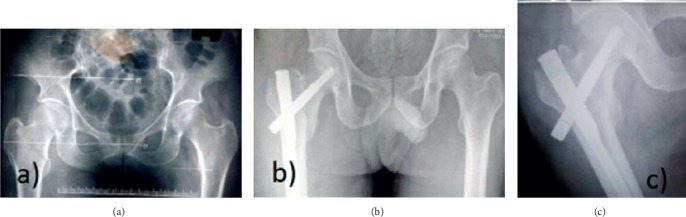
(a) Preoperative X-ray. (b) Immediate postoperative X-ray of PFNA-2 with lateral entry point showing lateral cortex impingement with gap at fracture site. (c) Follow-up at 6 months. Fracture union was seen at 6 months but with screw back out.

**Figure 5:**
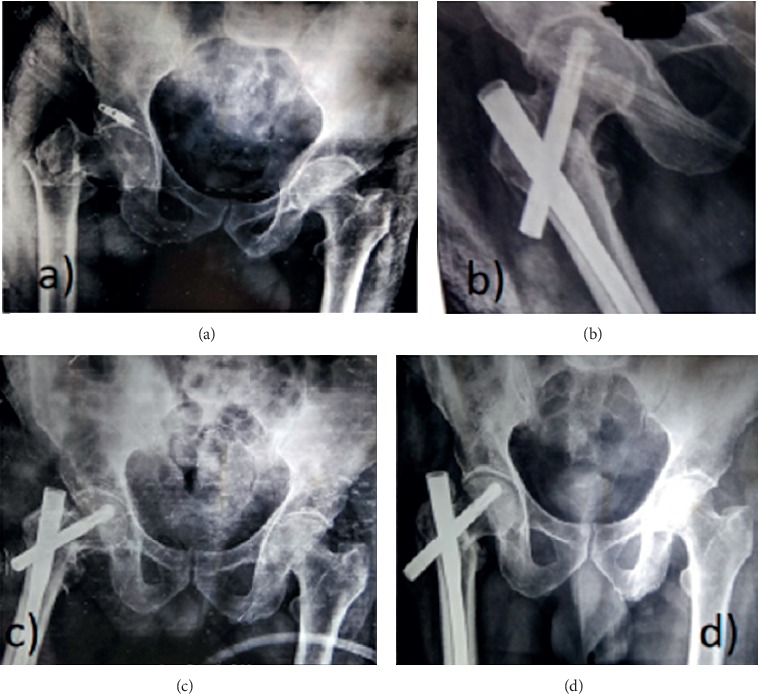
(a) Preoperative X-ray. (b) Immediate postoperative X-ray with lateral entry showing varus reduction, (c) at 6 weeks and (d) at final follow-up showing screw back out.

The lateral cortex impingement was seen in 14 patients of group L and 6 patients in group M with significant comparison (*P*=0.01) (Figure 4). Three patients in group L had varus collapse with screw back out and none in group M (*P*=0.05) (Figure 5). Screw back only was seen in one patient of group L. Subtrochanteric femur fracture was seen in 1 patient with lateral entry of the nail (Table 3). Out of three patients with varus collapse and screw back out, 2 were treated with exchange nailing and 1 with arthroplasty. The only case with screw back out was treated with implant removal (Figure 4).

### 3.3. Functional Outcome (Table 4 and Figure 6)

**Figure 6:**
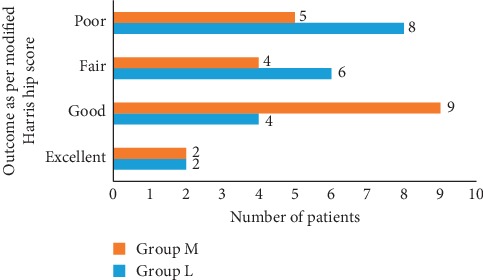
Functional outcome at 6 months.

The modified Harris hip score (average) for group L at 6 months was 71.94 and group M was 76.8 (*P*=0.84). In group L, excellent results were seen in 2 patients, 4 had good, 6 had fair, and 8 had poor results. In group M, 2 patients had excellent, 9 had good, 4 had fair, and 5 had poor results. When results of both the groups were correlated, there was no significance (*P*=0.84) (Table 4 and Figure 6).

## 4. Discussion

Entry point of the nail is more important as it will play a vital role in improving the quality of reduction and fixation and thus leading to good functional outcome without complications. The unstable intertrochanteric femur fracture in elderly needs good reduction and stable fixation, for early mobilisation [1]. Our aim was to figure out the optimal entry point for PFNA-2 which is widely being used at present in Asian population. There were no discrepancies in age distribution difference between the groups.

The tip apex distance was >25 mm in 4 patients with lateral entry. Three had screw back out. These 3 patients with screw back out had lateral cortex impingement. TAD was more than 25 mm in 5 patients in medial entry group and none showed any complications. There was no significance when TAD of both groups were compared. This supports the hypothesis of Kane et al. [16] that the position of the screw in the head and neck is more important than the tip apex distance.

This has also been concluded in the study by Nikoloski et al. [17] that a tip apex distance <25 mm is not a reliable indicator for PFNA. In their study on PFNA in elderly patients, Karapinar et al. [12] measured difference in neck shaft angle between surgically operated and normal hip. They found that 93% patients had good/acceptable reduction and 85.9% had ideal implant position. Out of 88% patients with neck shaft angle >120°, 12% had varus collapse. In a study by Radaideh et al. [18], 3 out of 4 patients with a neck shaft angle difference of more than 10° in the lateral entry group had screw back out. These three patients had varus reduction (<125°) of fracture which eventually led to loss of reduction and screw back out. The patients in the medial entry group had good/acceptable reduction as measured by the neck shaft angle. The reduction quality can be improved with entry point being just medial to the greater trochanter tip.

The ideal position of the screw was found to be in lower-centre and centre-centre position in the study by Kane et al. [16], and this resulted in stable fixation. As per the Cleveland index in present study, 4 out of 6 patients with suboptimal position in lateral entry group and none in medial entry group had screw back out. When results of both group were compared, there was no significance.

All three radiological parameters, when compared between the groups, had no significance. The complications were seen mainly in the lateral entry group and none in medial entry group. Hence, entry point of the nail is suggested to be the most important factor.

PFNA has been advised in elderly patients, and studies have found it to be a better implant and has given satisfactory functional and radiological results with minimal complications [18–20]. We got satisfactory results with PFNA-2, and there was no considerable difference between 2 groups in terms of functional outcome.

The varus collapse was seen in 12% [18], 5.8% [21], and 4.9% [22] of cases in previous studies. We got 15% cases with varus collapse with screw back out in the lateral entry group. There were no cases of varus collapse in the medial entry group. PFNA-2 had minimized lateral cortex impingement in unstable peritrochanteric fractures as concluded in a study by Macheras et al. [23]. We encountered lateral cortex impingement in 70% patients of group L and 30% patients of group M with unstable intertrochanteric femur fracture. Nail shaft axis was described by Jiamton et al. as a potential risk factor for failed osteosynthesis due to its association with secondary varus displacement [24]. Tao et al. [25] emphasized that regardless of the implant choice and its characteristics, the inserting technique is the key factor for stable fixation without complications. Hence, we recommend the entry point for PFNA-2 should be 5 mm medial to the greater trochanter tip for achieving adequate fixation and thus minimizing complications. More damage to the gluteus medius insertion has been described by McConnell et al. [26]. An average of 27% tendon damage might occur during reaming of entry point which could be a cause of postoperative morbidity. The placement of the trochanteric entry point is difficult to precisely locate intraoperatively by image intensifier. High degree of variability existed with respect to trochanteric entry point according to Streubel et al., and they concluded preoperative templating was an accurate way of obtaining ideal entry point [27]. The limitation of our study was short follow-up period of six months.

## 5. Conclusion

Both the entry points gave equivocal functional results at final follow-up (*P*=0.8445). More complications were encountered with lateral entry point compared to medial entry (*P*=0.05). The lateral entry point showed more cases with lateral cortex impingement as compared to medial entry (*P*=0.01). The outcome can be good when the TAD is less than 25 mm, neck shaft angle difference is less than 5°, and the Cleveland index is in an optimal position (centre-centre or inferior-centre). Overall, to achieve good quality of fixation and minimal damage to the gluteus medius, the entry point for PFNA-2 should be 5 mm medial to the greater trochanter tip.

## Figures and Tables

**Table 1:** Demographic statistics.

*n* = 40	Group L (20)	Group M (20)	*P* value
Age (average)	69.6	69.85	0.23

Sex			
Male	8	12	0.1
Female	12	8	

Fracture type			
31. A2	13	11	0.37
31. A3	7	9	

**Table 2:** Radiographic parameters.

*n* = 40	Group L (20)	Group M (20)	*P* value
Tip apex distance (more than 25 mm)	4	5	0.5

Average tip apex distance	20.53	20.02	0.8
Mean and SD	6.2	6.63	

Neck shaft angle (difference between operated and normal side)			
<5° good	13	14	0.08
5°–10° acceptable	3	6	
>10° poor	4	0	

Cleveland index			
Suboptimal position	6	6	0.63
Ideal position	14	14	

**Table 3:** Complications.

*n* = 40	Group L (20)	Group M (20)	*P* value
Varus collapse + screw back out	3	0	0.05
Screw back out	1	0	

Lateral cortex impingement	14	6	0.01
Subtrochanteric fracture	1	0	

**Table 4:** Functional outcome at 6 months.

*n* = 40	Group L (20)	Group M (20)	*P* value
Modified Harris hip score at six months	71.94	76.8	0.84
(Mean and SD)	14.81	14.15	

## Data Availability

Data will be provided on request.
